# Exploring Correlations of Food-Specific Disgust with Eating Disorder Psychopathology and Food Interaction: A Preliminary Study Using Virtual Reality

**DOI:** 10.3390/nu15204443

**Published:** 2023-10-19

**Authors:** Sevgi Bektas, Ludovica Natali, Katie Rowlands, Lucia Valmaggia, Jerome Di Pietro, Hiba Mutwalli, Hubertus Himmerich, Janet Treasure, Valentina Cardi

**Affiliations:** 1 Centre for Research in Eating and Weight Disorders (CREW), Institute of Psychiatry, Psychology and Neuroscience, King’s College London, London SE5 8AF, UK; katie.rowlands@kcl.ac.uk (K.R.); hiba.mutwalli@kcl.ac.uk (H.M.); hubertus.himmerich@kcl.ac.uk (H.H.); janet.treasure@kcl.ac.uk (J.T.); valentina.cardi@unipd.it (V.C.); 2 Department of Psychology, Hacettepe University, Ankara 06800, Türkiye; 3 Department of General Psychology, University of Padova, 35122 Padova, Italy; 4 Department of Psychology, Institute of Psychiatry, Psychology & Neuroscience, King’s College London, London SE5 8AF, UK; lucia.valmaggia@kcl.ac.uk (L.V.); jerome.dipietro@kcl.ac.uk (J.D.P.); 5 South London and Maudsley NHS Foundation Trust, London SE5 8AB, UK; 6 Department of Psychiatry, KU Leuven, 3000 Leuven, Belgium; 7 Department of Clinical Nutrition, College of Applied Medical Sciences, Imam Abdulrahman Bin Faisal University, Dammam 34212, Saudi Arabia

**Keywords:** anorexia nervosa, disgust, food, eye gaze, touch, virtual reality

## Abstract

The emotion of disgust is thought to play a critical role in maintaining restrictive eating among individuals with anorexia nervosa. This exploratory cross-sectional study examined correlations between food-specific trait and state disgust, eating disorder psychopathology, illness severity (body mass index: BMI), and interactions with virtual foods in people with anorexia nervosa. Food-specific trait disgust and eating disorder symptoms were measured before exposure to virtual foods in one of three virtual reality (VR) kitchens to which participants were randomly allocated. Food interactions (eye gaze and reaching towards virtual foods) were measured during the VR exposure. Food-specific state disgust ratings were collected after the VR exposure. In the entire sample, eating disorder symptoms correlated positively with food-specific trait disgust (rs (68) = 0.45, *p* < 0.001). We also found a significant association between food-specific state disgust and eating disorder symptoms in each virtual kitchen scenario: virtual kitchen only (rs (22) = 0.40, *p* = 0.05), virtual kitchen plus pet (rs (22) = 0.80, *p* < 0.001), and virtual kitchen plus avatar (rs (20) = 0.78, *p* < 0.001). No significant correlation was observed for the link between food-specific disgust measures and food-related touch. Correlations between food-specific trait disgust and food-related eye gaze differed across scenarios. The current experimental paradigm needs to be improved to draw firm conclusions. Both food-specific trait and state disgust are associated with eating disorder psychopathology, and therefore, effective strategies are warranted to attenuate food-specific disgust.

## 1. Introduction

Anorexia nervosa (AN) is a psychiatric disorder defined by a persistent and pervasive tendency of food avoidance. Exposure to food- or eating-related cues is associated with expected negative consequences (e.g., losing control, weight gain) and aversive emotions [1]. Much of the literature in this field has focused on the emotions of fear and anxiety, whereas only a few studies have investigated the role of disgust in maintaining eating disorder behaviours. A recent systematic review and meta-analysis of disgust in eating disorders [2] indicated that disgust in response to high-calorie food images was significantly elevated in people with eating disorders, whereas generic disgust sensitivity was lower. Individual differences in food disgust sensitivity compared to generic disgust sensitivity may be a more salient concept in people with AN since they tend to report disgust towards specific foods or food characteristics. Previous studies have examined state disgust, such as disgust reactions in response to food pictures in AN [2]. However, a pervasive pattern of food-specific disgust as a potential trait of individuals with AN has not yet been the focus of research.

Both state and trait forms of food-specific disgust may play a key role in shaping cognitive and behavioural mechanisms underlying AN symptomatology. For example, attentional or behavioural bias for food in AN has been studied using behavioural tasks and eye-tracking paradigms [3,4]. A recent systematic and methodological review of this literature [5] highlighted the variations in paradigms or stimuli used across studies. These variations have limited researchers’ ability to conclude on the direction of biases (hypervigilance or avoidance of the stimuli). Nevertheless, it has been reported that attentional and/or behavioural avoidance of food, especially foods with high fat content, may be a common phenomenon in AN. It might be also possible that people with AN who experience greater food disgust may show enhanced attention and preparedness or greater avoidance of certain information or signals associated with danger (e.g., foods of unknown caloric density or changes in appearance), as suggested in the vigilance and avoidance model of attentional bias [6,7].

It is critical to explore relevant attentional and behavioural patterns linked to food disgust and eating disorder symptoms in AN for the development of treatment plans targeting disgust as well as anxiety or fear. Recently, virtual reality has been utilised as a practical research tool in the assessment of food-related attentional and behavioural mechanisms—for example, in people with binge eating disorders [8]. However, to our knowledge, data on disgust in AN using this technology are non-existent. Hence, the aim of this research is to test correlations between food-specific disgust, eating disorder psychopathology, and food interaction across different virtual kitchen scenarios in people with AN. No specific hypotheses were formulated due to the exploratory nature of this study.

## 2. Materials and Methods

### 2.1. Participants

English-speaking participants under treatment for AN were recruited from South London and Maudsley NHS Foundation Trust, through social media, and via email circulars at King’s College London (KCL) between September 2022 and March 2023. The exclusion criteria were (1) age under 14 years; (2) body mass index (BMI) > 25; (3) self-reported diagnosis of neurological disorders, psychosis, or substance abuse disorders; and (4) visual/hearing impairments not corrected by glasses/hearing aids. All participants provided informed consent. The study was approved by the Research Ethics Committee North West—Liverpool East (reference number: 18/NW/0853) in the UK.

### 2.2. Apparatus

The virtual reality (VR) environment was developed by the KCL VR Research Lab for an ongoing large study led by V.C. and supported by the Medical Research Council [9]. The VR environment was built using the Unity3D game engine, Oculus Integration SDK for Unity, and Oculus Quest 2 headsets. A head-mounted display (HMD) headset and two Oculus Touch controllers were used. The headset has six degrees of freedom (6DoF) technology, which provides continuous rotational and positional tracking. The screen had a resolution of 1832 × 1920 pixels per eye.

The five-minute 3D scenario displayed a virtual kitchen, which stored foods of various calorie contents. A full list of the virtual foods is provided in Table S1. Three different versions of the virtual kitchen scenario were used: (1) a virtual kitchen only, (2) a virtual kitchen plus a virtual pet to induce a positive mood, and (3) a virtual kitchen plus an avatar to induce social support (see Text S1). Pre-recorded vocal instructions were used to guide participants on freedom to move in the kitchen, to look around, to open the fridge/cupboards, and to reach out to touch and hold the food using the hand controllers (for pictures of the virtual kitchen environment, see Figure S1).

### 2.3. Procedure

After providing written consent, participants completed a Qualtrics survey consisting of demographic and baseline questionnaires (related to eating disorder psychopathology and disgust sensitivity). Subsequently, participants were invited to complete the VR exposure session at KCL. During their lab visit, they were individually guided to familiarise themselves with the Oculus Quest 2 headset and controllers in a quiet room and then entered one of three virtual kitchen scenarios selected at random for each participant.

The use of components that increase the presence of positive emotional experiences can be beneficial for psychological treatment adjuncts [10]. So far, it has been demonstrated that both positive mood induction and recovery-focused support from peers are associated with increased calorie intake during a standard test meal and decreased attention bias to food after the test meal compared to a control condition [11,12,13]. In addition, it has been reported that encouraging support from people with lived experiences is highly endorsed by patients over time and associated with fewer eating disorder symptoms [12]. Based on this evidence, a positive mood induction condition and a social support condition were combined with the food exposure in the virtual kitchen scenarios with the goal of exploring whether these forms of support might impact participants’ emotions/behaviours in the virtual kitchen compared to the food-only condition.

In the virtual kitchen plus pet scenario, participants entered the kitchen with a virtual pet, a pink elephant making soft gurgling noises and following the participant around the environment. In the virtual kitchen plus avatar scenario, participants were accompanied in the kitchen by a virtual avatar (they could choose between four different avatars, see Figure S2) who spoke from a supportive and motivational script (e.g., “It is normal to feel anxious, you are doing the best you can right now. Nobody is here to judge what you will do in the kitchen, which foods you will look at or grab. Take a few deep breaths, slow your mindset down, and think of the bigger picture. Each minute more is a minute easier”), encouraging participants to challenge the eating disorder voice and approach the virtual foods.

After the completion of the exposure session, participants were asked to rate their disgust at the thought of eating the high-calorie or palatable foods that were shown in the virtual kitchen. Participants were debriefed and reimbursed with a GBP 20 Amazon voucher upon completing the survey and VR exposure.

### 2.4. Measures

The following data were collected:

Before VR exposure

The socio-demographic and clinical variables collected were age, gender, ethnicity, years of education, illness duration, self-reported weight, and height (used to calculate BMI as kg/m^2^). At baseline, participants also completed the Food Disgust Scale [14], a 32-item scale measuring eight domains of food disgust (Cronbach’s alpha in this study = 0.93) as a parameter reflecting food-specific trait disgust, and the Eating Disorders Examination Questionnaire [15], a 28-item scale for the assessment of eating disorder symptom severity (Cronbach’s alpha in this study = 0.94), with four dimensions: dietary restraint, shape concern, weight concern, and eating concern.

During VR exposure

**Figure 1:**
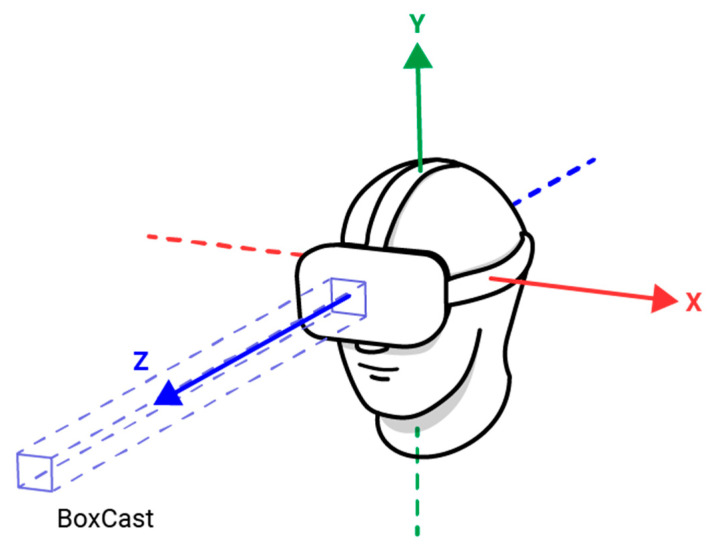
A diagram of the virtual reality device.

The frequency of eye gazes towards and touching of virtual foods were automatically recorded in the VR environment by the software. The application used a narrow BoxCast, a rectangular ray fired along the Quest HDM’s *z*-axis (see Figure 1, produced by J.D.P.), to approximate eye gaze direction. The head movement can, in this way, be used to determine where the participant is looking at the task, despite its limitation to perfectly analogous and accurate eye tracking.

After VR exposure

As a parameter reflecting state disgust, momentary disgust reactions towards pictures of high-calorie foods that were shown in the virtual kitchen were collected using a Likert scale ranging from 0 (not at all) to 7 (extremely disgusted) with one question (“How disgusted do you feel now about eating high-calorie or palatable foods (e.g., pizza, chocolate, crisps)?”).

For a summary of measures collected at different time points, see Table 1.

### 2.5. Statistical Analyses

All statistical analyses were performed with SPSS v.28 (IBM Corp., Armonk, NY, USA). The descriptive analyses provided an overview of the demographic and clinical variables of the entire sample. Data were non-parametrically distributed. Thus, median and interquartile ranges were reported. The Pearson Chi-square test and the Kruskal–Wallis one-way analysis of variance with a post hoc Dunn test was used to compare the participants’ characteristics among different virtual kitchen scenarios since the criteria for homogeneity of variance was not met. Spearman rank–order correlation coefficients were used to identify the strength and directions of relationships between the variables of interest. Significance was determined with *p*-values ≤ 0.05. We did not control multiple measurements, as this was an exploratory study.

## 3. Results

A total of 70 participants with AN with an average age of 25.7 (SD = 7) years completed the study. Most participants were female (94.3%) and White British (88.6%). The overall mean BMI was 16.8 (SD = 2.5) kg/m^2^.

The Kruskal–Wallis test revealed significant differences between the three virtual scenarios only for illness duration. A follow-up Dunn test revealed that the length of illness duration of participants in the virtual kitchen plus avatar scenario was significantly lower than those in the kitchen-only scenario (H (2) = 7.91, *p* = 0.02). Descriptive statistics and group comparisons are reported in Table 2.

### 3.1. Correlations between Eating Disorder Psychopathology and Food-Specific Disgust Measures

The associations between food disgust sensitivity (food-specific trait disgust), post-exposure disgust towards high-calorie foods alone (food-specific state disgust), eating disorder severity (EDE-Q global and BMI), and food interactions (eye gazes towards and touching virtual foods) are presented in Table 3. The SPSS output for Spearman rank-order correlations and scatterplots are available in the Supplementary Materials (see Figures S3 and S4).

In the entire sample, the correlation between food disgust sensitivity (food-specific trait disgust) and the EDE-Q global score was 0.45 (*p* < 0.001, 95% CI [0.24, 0.63]), indicating a moderate relationship. There were no statistically significant correlations of food-specific disgust measures and BMI.

The correlation between post-exposure disgust ratings (food-specific state disgust) and the EDE-Q global scores was also significant in each virtual kitchen scenario: virtual kitchen only (rs = (22) = 0.40, *p* = 0.05, 95% CI [−0.02, 0.70]), virtual kitchen plus pet (rs = (22) = 0.80, *p* < 0.001, 95% CI [0.56, 0.92]), and virtual kitchen plus avatar (rs = (20) = 0.78, *p* < 0.001, 95% CI [0.52, 0.92]). However, there were no statistically significant correlations between post-exposure disgust ratings (food-specific state disgust) and BMI in the three virtual kitchen scenarios.

### 3.2. Correlations between Food Interaction and Food-Specific Disgust Measures in the Virtual Kitchen Scenarios

Food disgust sensitivity (food-specific trait disgust) was significantly and positively correlated with frequency of eye gazes towards virtual foods (rs = (22) = 0.66, *p* < 0.001, 95% CI [0.34, 0.84]) in the virtual kitchen plus pet scenario. There were no statistically significant correlations between food-specific disgust measures and food interaction measures in the virtual kitchen-only scenario or in the virtual kitchen plus pet condition.

## 4. Discussion

This explorative study assessed correlations between food-specific trait and state disgust, eating disorder psychopathology (EDE-Q global and BMI), and food interaction measures (eye gaze and touch) in people with AN. We collected food disgust sensitivity (trait disgust) and eating disorder psychopathology data at baseline before participants were randomly assigned to the different virtual kitchen scenarios in which food interaction measures were obtained. Participants were then asked to rate their disgust reactions (state disgust) to high-calorie foods. The correlations involving variables collected during or after the VR paradigm were examined for each scenario rather than the whole sample. There are two main findings in the present study. The first finding is that food-specific trait and state disgust were significantly and positively associated with eating disorder symptom severity. The second finding is that inconsistent correlations between food disgust sensitivity and food-related eye gaze in different virtual kitchen scenarios were observed.

The first finding of an association between trait and state disgust with eating disorder psychopathology can be interpreted as an association of eating disorder severity with food-specific disgust. This is in line with a recent systematic review and meta-analysis that found that the levels of generic disgust sensitivity, self-disgust sensitivity, and momentary disgust to food images were significantly higher in people with eating disorders than in healthy controls [2]. Food disgust sensitivity has recently been investigated in non-clinical populations, and findings indicate that food disgust sensitivity may potentially influence food- or eating-related behaviours (e.g., texture-based food rejection, picky eating, selective eating, and variety-seeking in food) [16,17]. Surprisingly, there has been no research in patients with eating disorders so far in this regard.

In addition, food-specific state disgust correlated with eating disorder severity in the three different virtual kitchen scenarios. However, there was no difference among the scenarios regarding the question of “How disgusted do you feel now about eating high-calorie or palatable foods (e.g., pizza, chocolate, crisps)?” This indicates that there is no post-exposure effect on disgust ratings. However, this question is not suitable for investigating the impact of the kitchen scenarios comprehensively on the food-specific state of disgust. In addition, the experimental paradigm was primarily designed to test whether induced social support or positive mood would enhance the impact of virtual food exposure on food-related anxiety in people with AN [9]. It has been reported that disgust is more resistant to change compared to anxiety and fear [18], and therefore the development of treatment strategies directly targeting disgust in response to food or eating is necessary. Improvements in the current VR paradigm could be considered to ameliorate food-specific state disgust in the future. The availability of compassionate social support and social networks has a key role in the treatment of individuals with AN in terms of developing new positive associations with food stimuli [19]. Thus, an exposure to food-related environments providing social contact in VR settings may motivate individuals with AN to approach disgust-eliciting foods. For example, not only the company of the virtual avatar but also a positive interaction/dialogue in the virtual kitchen may have a potential therapeutic value of improving positive emotions (e.g., joy, connectedness, optimism) and diminishing disgust. Developing tailored and personally relevant scripts addressing disgust feelings in response to high-calorie foods and relevant negative expectations (e.g., body signals, taste pleasantness, or weight gain) and motivations (e.g., avoidance) might be useful.

Our second finding suggests that correlations between food disgust sensitivity and food-related eye gaze differed across the three virtual kitchen scenarios. A positive correlation between food disgust sensitivity and food-related eye gaze was observed for people with AN in the virtual kitchen plus pet scenario. Interestingly, this relationship was non-existent in the other two conditions. The variations between the conditions might partially explain the discrepancies in the results. Specifically, participants in the virtual kitchen plus avatar condition had a higher BMI and lower illness duration in comparison with those in the virtual kitchen plus pet condition. Lower BMI and longer duration of eating disorder diagnosis have previously been associated with non-response to treatment [20] or worse short-term treatment outcomes [21] in people with AN. The relationship between food disgust sensitivity and eye gaze towards virtual food items might be more evident in people with greater eating disorder severity. On the other hand, if the illness duration is critical to seeing a statistically significant correlation, the absence of this relationship in the kitchen-only scenario for participants with the longest illness duration is puzzling. This raises the question of whether the presence of a pet might motivate people with greater food disgust sensitivity to look at food items more often in the virtual kitchen environment. The inconsistent findings might also be explained by the low power due to the small sample size across the virtual kitchen scenarios. The correlation of food-specific trait and state disgust with food interaction measures should be tested in a larger sample of AN patients to better understand the relationship between food disgust sensitivity and food-related eye gaze. To prevent variations between conditions for illness duration, stratified randomisation may be considered.

## 5. Clinical Implications

In order to draw clinical conclusions, longitudinal studies on the relevance of disgust for the development of eating disorder pathology are needed. If this causal connection is confirmed, then strategies that enable people to modify their perception of food-specific disgust could be useful for the prevention and treatment of AN and other eating disorders.

So far, most interventions for AN have focused on food-related fears rather than disgust around food or eating. Our study highlights the importance of targeting food-specific disgust as well. Behavioural strategies—for example, exposure and counterconditioning of US revaluation—are recommended to reduce extinction-resistant disgust responses in the literature on obsessive-compulsive disorders [18]. Future research could test whether increasing the frequency and duration of exposure-based techniques leads to more effective and longer-term effects in reducing disgust-based avoidance. Cognitive techniques may also be of use to evaluate thoughts related to their perceptions of unpleasant or threatening experiences of food-specific disgust.

## 6. Strengths and Limitations

This study has two main strengths. First, to the best of our knowledge, this is the first study to examine the correlation between food-disgust sensitivity and eating disorder symptom severity in people with AN. Second, the VR paradigm enabled aspects of attention and approaches towards food to be measured.

There are some limitations in the present study to acknowledge. The first limitation is the lack of a healthy control group. Including a healthy control group would be valuable for supporting the hypothesis that food-specific disgust is critical to target in the treatment of anorexia and when investigating aberrant patterns in food-related eye gaze and touch. The second is the smaller number of participants from ethnic minority backgrounds, limiting the generalisability. Possible explanations could be that young White females, the majority of the participants, have more cultural pressure on body image, greater awareness of the illness, or access to better medical care [22,23]. Further studies may have a healthy control group and may implement new recruitment strategies to increase ethnic diversity in clinical anorexia research [24]. Future studies could also examine differences between individuals with diverse types of food-related avoidance (e.g., avoidant–restrictive food intake disorder, restrictive and binge–purge-type AN) to address important research questions of clinical significance. In the current sample, autistic traits were not evaluated. However, in patients with AN who have autistic traits, expectation about the impact of food on the body with aversive feelings or physical sensations appear to be more frequent and stronger than in non-autistic patients and more likely to generalise to other foods. Elevated food disgust sensitivity may cause susceptible individuals to experience disgust when encountering combined or modified edible foods (e.g., bread with seeds, wonky strawberries, chocolate with nuts) in a similar way to encountering inedible foods. Thus, having participants who are more similar (e.g., individuals with both AN and autism spectrum traits) might be useful in minimising the within-subject variability and being able to evaluate the effects of different virtual kitchen scenarios. Improving the existing food list with the more complex aforementioned foods could help with the assessment of food disgust sensitivity for people with more complex sensory difficulties related to food (e.g., some autistic individuals).

The current experimental paradigm also has limitations. For example, food-specific trait and state disgust were measured at different time points. Only one question was utilised to measure disgust reactions to a group of high-calorie foods that were shown in the virtual kitchen. Food-specific state disgust responses collected before and after the virtual kitchen exposure via VR would help to evaluate the effects of different virtual kitchen scenarios. Exposure to a virtual kitchen with a wider range of foods (e.g., decaying melon, wrinkled tomato, chocolate with chocolate bloom, snails, oysters, and grasshoppers) might be more likely to elicit disgust, especially in people with a greater level of food disgust sensitivity. To further increase the sense of presence in VR and to make the user experience even more immersive, it might be useful to include more sensory modalities that occur during interaction with food, such as tactile feedback and smell. With regards to food interaction measures, it might be useful to analyse them specifically in relation to the calorie content of virtual food items. In addition to the VR touch feature, other measures of food avoidance could be tested in a larger sample to draw firm conclusions regarding the link between food-related touch and food-specific disgust.

## 7. Conclusions

The present explorative study tested the correlations of food-specific disgust measures with eating disorder psychopathology and food interaction measures by using the VR paradigm in people with AN. The findings showed that both food-specific trait and state disgust were correlated with eating disorder severity. The correlation between food-specific trait disgust and food-related eye gaze was statistically significant in the virtual kitchen plus pet scenario, but not in the other two virtual kitchen scenarios. The inconsistent findings could be explained by the small sample size or the effect of variations in the virtual kitchen scenarios. The present study offers valuable insight into ways to modify the design of future VR studies to examine potential correlates of food-specific disgust in people with AN, which could contribute to assessment and treatment strategies.

## Figures and Tables

**Table 1:** Summary of measures collected in the present study.

**Before the VR kitchen**	**During the VR kitchen** (Participants were randomly allocated to different scenarios: (1) virtual kitchen only, (2) virtual kitchen + pet, (3) virtual kitchen + avatar	**After the VR kitchen**
**Food-specific trait disgust measure:** Food Disgust Scale, a 32-item scale from 1 (not disgusting at all) to 6 (extremely disgusting) for food disgust sensitivity.	**Food interaction measure:** The frequency of food-related eye gaze towards virtual food items.	**Food-specific state disgust measure:** A Likert scale with one question (“How disgusted do you feel now about eating high-calorie or palatable foods (e.g., pizza, chocolate, crisps)?”) for post-exposure disgust towards high-calorie foods that were shown in the virtual kitchen.
**Eating disorder psychopathology measure:** Eating Disorder Examination Questionnaire, a 28-item scale for eating disorder symptom severity.	**Food interaction measure:** The frequency of food-related touching of virtual food items.	
**Illness severity measure:** body mass index (BMI) as kg/m^2^		

**Table 2:** Descriptive statistics of the demographic and clinical variables and group comparisons.

	N (%) or Median (IQR)				
Variable	Whole Sample (*n* = 70)	Kitchen (*n* = 24)	Kitchen + Pet (*n* = 24)	Kitchen + Avatar (*n* = 22)	Test of Group Differences
Age	23.5 (21.00–29.25)	26.0 (21.00–29.75)	24.5 (16.0–40.0)	22.5 (20.00–26.25)	H (2) = 3.15, *p* = 0.21
Sex					Χ2 (4) = 6.17, *p* = 0.19
Female	66 (94.3%)	21 (87.5%)	24 (100%)	21 (95.5%)	
Male	2 (2.9%)	1 (4.2%)	N/A	1 (4.5%)	
Other	2 (2.9%)	2 (8.3%)	N/A	N/A	
Ethnicity					Χ2 (6) = 10.42, *p* = 0.11
Asian British	2 (2.9%)	2 (8.3%)	N/A	N/A	
White British	62 (88.6%)	19 (79.2%)	22 (91.7%)	21 (95.5%)	
Mixed race	2 (2.9%)	N/A	1 (4.2%)	1 (4.5%)	
Other (non-British)	4 (5.7%)	3 (12.5%)	1 (4.2%)	N/A	
Years of education	16.0 (14.00–18.00)	16.0 (15.00–18.00)	15.5 (13.00–18.00)	16.0 (14.00–18.00)	H (2) = 0.24, *p* = 0.89
Illness duration	8.0 (4.75–12.00)	10.0 (7.13–13.00)	8.0 (2.88–13.75)	6.5 (3.50–9.00)	H (2) = 7.91, *p* = 0.02 * Kitchen > kitchen + avatar, p = 0.02
BMI (kg/m2)	16.6 (15.34–18.47)	16.5 (14.90–18.24)	16.5 (14.96–17.64)	17.3 (17.29–19.61)	H (2) = 3.40, *p* = 0.18
EDE-Q					H (2) = 0.25, *p* = 0.88
EDE-Q global	3.6 (2.72–4.73)	3.8 (2.92–4.84)	3.5 (2.50–4.72)	4.0 (2.21–4.88)	
EDE-Q restraint	3.9 (2.35–4.65)	3.7 (2.10–4.75)	3.9 (2.45–4.60)	4.2 (2.00–4.85)	
EDE-Q eating concern	2.6 (1.55–4.20)	2.8 (1.80–4.70)	2.6 (1.45–3.70)	2.3 (1.30–4.40)	
EDE-Q weight concern	4.0 (2.80–5.40)	4.1 (2.90–5.40)	4.0 (3.00–5.20)	4.0 (2.50–5.40)	
EDE-Q shape concern	4.5 (3.35–5.63)	4.6 (3.53–5.60)	4.4 (3.38–5.38)	4.8 (2.88–5.91)	
FDS					H (2) = 0.05, *p* = 0.98
FDS global	4.0 (3.27–4.69)	4.0 (3.30–4.85)	4.2 (3.10–4.71)	3.8 (3.30–4.53)	
FDS animal meat	4.8 (3.50–5.50)	4.9 (3.38–5.94)	4.9 (3.56–5.50)	4.5 (3.44–5.31)	
FDS poor hygiene	5.6 (4.95–6.00)	5.8 (4.85–6.00)	5.5 (4.45–5.95)	5.7 (5.30–5.85)	
FDS human contamination	3.0 (1.94–4.50)	3.4 (2.00–4.50)	3.5 (1.81–6.00)	2.6 (1.75–3.63)	
FDS mold	4.0 (4.00–5.50)	4.0 (2.06–5.25)	3.9 (1.81–6.00)	4.9 (2.38–5.50)	
FDS decaying fruit	2.5 (1.25–4.00)	2.5 (1.31–4.00)	2.1 (1.38–4.00)	2.9 (1.19–3.75)	
FDS fish	3.4 (2.25–5.00)	3.3 (2.50–5.50)	3.8 (1.81–5.00)	3.3 (2.25–4.31)	
FDS decaying vegetables	3.4 (2.25–4.56)	3.0 (2.25–4.19)	3.4 (2.25–4.44)	4.1 (2.81–4.75)	
FDS living contamination	6.0 (4.92–6.00)	6.0 (5.08–6.00)	6.0 (4.67–6.00)	5.7 (4.92–6.00)	
Food interaction					
VR eye gazes	290.5 (229.50–353.25)	287.0 (233.25–379.75)	292.0 (216.75–357.75)	292.5 (216.50–347.00)	H (2) = 0.03, *p* = 0.99
VR touches	19.0 (12.00–25.25)	21.5 (15.00–29.75)	18.5 (12.00–22.00)	15.0 (9.50–26.50)	H (2) = 5.26, *p* = 0.07
Post-VR disgust	5.0 (4.00–6.00)	5.0 (4.00–6.00)	6.0 (4.00–6.50)	5.0 (3.00–6.25)	H (2) = 0.36, *p* = 0.84

Abbreviations. IQR, interquartile range; N/A, not applicable; BMI, body mass index; EDE-Q, Eating Disorder Examination Questionnaire; FDS, Food Disgust Scale; VR eye gazes, the frequencies of eye gazes towards virtual foods; VR touches, the frequencies of touching virtual foods; post-VR disgust, momentary disgust reactions towards virtual foods with high-calorie content measured following the VR exposure; H, Kruskal–Wallis test; X^2^, Pearson Chi-square test. * *p* ≤ 0.05.

**Table 3:** Spearman rank–order correlations for variables of interest in people with anorexia nervosa.

VR Scenarios	Variables					
	I FDS_Global	II Post-VR Disgust	III EDE-Q Global	IV BMI	V VR Eye Gazes	VI VR Touches
**All Sample (*n* = 70)**						
I FDS global	1.00					
II Post-VR-Disgust	0.16	1.00				
III EDE-Q global	0.45 **	0.66 **	1.00			
IV BMI	0.15	−0.01	−0.00	1.00		
V VR eye gazes	0.25 *	−0.13	−0.16	0.27 *	1.00	
VI VR touches	−0.22	−0.17	−0.35 **	−0.07	0.33 **	1.00
**Kitchen (*n* = 24)**						
I FDS global	1.00					
II post-VR-disgust	0.14	1.00				
III EDE-Q global	0.53 **	0.40 *	1.00			
IV BMI	0.05	−0.04	0.02	1.00		
V VR eye gazes	0.11	−0.27	−0.33	0.12	1.00	
VI VR touches	−0.36	−0.14	−0.31	−0.36	0.33	1.00
**Kitchen + Pet (*n* = 24)**						
I FDS global	1.00					
II post-VR disgust	0.16	1.00				
III EDE-Q global	0.33	0.80 **	1.00			
IV BMI	0.10	−0.36	−0.42 *	1.00		
V VR eye gazes	0.66 **	0.17	0.09	0.35	1.00	
VI VR touches	0.15	−0.15	−0.47 *	0.34	0.37	1.00
**Kitchen + Avatar (*n* = 22)**						
I FDS global	1.00					
II post-VR disgust	0.19	1.00				
III EDE-Q global	0.45 *	0.78 **	1.00			
IV BMI	0.29	0.24	0.28	1.00		
V VR eye gazes	0.02	−0.26	−0.23	0.25	1.00	
VI VR touches	−0.33	−0.20	−0.40	−0.21	0.28	1.00

Abbreviations. IQR, interquartile range; BMI, body mass index; EDE-Q, Eating Disorder Examination Questionnaire; FDS, Food Disgust Scale; VR eye gazes, the frequency of eye gazes towards virtual foods; VR touches, the frequency of touching virtual foods; post-VR disgust, momentary disgust reactions towards virtual foods with high-calorie content measured following the VR exposure. * *p* ≤ 0.05; ** *p* < 0.001.

## Data Availability

The data that support the findings of this study are available from the corresponding author, S.B., upon reasonable request.

## Supplementary Materials

The following supporting information can be downloaded at the following link: https://www.mdpi.com/article/10.3390/nu15204443/s1, Table S1: List of virtual foods; Text S1: Script used in the kitchen + avatar scenario; Figure S1: Pictures of the virtual kitchen environment; Figure S2: Selection of avatars available to participants; Figure S3: SPSS output for spearman rank–order correlations; Figure S4: Scatter plot figures.
