# Supplementary material for: Exploring Correlations of Food-Specific Disgust with Eating Disorder Psychopathology and Food Interaction: A Preliminary Study Using Virtual Reality
Source: Nutrients. 2023 Oct 19;15(20):4443. doi: 10.3390/nu15204443 (PMC10609698; doi:10.3390/nu15204443)

Table S1. The list of virtual foods

The description of food items with different calorie content in the virtual kitchen scenarios

Apple	Orange	Pizza	Pirate charms
Black tea	Pear	Raspberry	Summer berry crisp
Lager beer	Pineapple	Siracha	Wheat crunchies
Bread	Canned baked beans in tomato sauce	Spaghetti Bolognese	Original chips
Toast bread	Canned baked beans	Sushi plate with soy	Cane sugar
Chocolate chip cookie	Canned garden peas	White toast	Cookie crumby
Chocolate granola bar	Canned tinned tomato	Canned chicken soup	Salt crackers
Coffee beans	Canned tuna chunks	Cup noodle	Bubble gum mega big
Croissant	Fusilli dried pasta	Macaroni and cheese	Dark chocolate
Draft beer	Whole penne pasta	Wheat flour	Lollipops
Egg sandwich	Vanilla filled cake	Chocolate syrup	Broccoli
Crunch crisps	Cheese	Whipped cream	Cabbage
Chicken nuggets	Crème Fraiche	Lemon sparkly drink	Red bell pepper
Chocolate ice cream	Full-fat mayo	Tomato ketchup	Coffee
Chocolate chip ice cream	Packed apple juice	Yellow mustard	Fried eggs with bacon
Frozen chips	Large eggs	Raw sushi rolls	Green soup
Low fat strawberry yoghurt	Lightly salted butter	Red bell pepper	Pepperoni
	Low fat milk	Carrot	Peanut butter
		Whole milk	Fish fingers

Text S1. The script used in the virtual scenario: Kitchen + Avatar.

You are likely to experience the tricks and torments of the eating disorder voice in this moment. The voice of people who recovered from the illness might be of great help in these difficult moments. The words I will share are their words.

It is normal to feel anxious, you are doing the best you can right now. Nobody is here to judge what you will do in the kitchen, which foods you will look at, or grab.

Take a few deep breaths, slow your mindset down and think of the bigger picture. Each minute more is a minute easier.

While challenging the eating disorder thoughts, remind yourself of all that you have achieved so far, the relationships you share, the passions and dreams you have, they are so important and will ultimately help you to break free of the eating disorder constraints. Focus on your skills and strengths right now. What would other people say are your skills and strengths? 3-second pause

Perhaps you are someone who, despite the setbacks and difficulties experienced in the past, is still fighting for a happier life? Perhaps you have helped others in the past, and could use some of the same strategies to encourage yourself in this moment? 3-second pause

Everyone else deserves good and balanced nutrition. Everyone else deserves to have a good relationship with food. What makes you different?

Food is not the enemy, think of it as nourishment for your body and food for your soul. Food provides the energy and motivation to allow you to perform at your best. Food is our medicine, and we cannot live without it.

The eating disorder might be speaking louder right now, but you are stronger than that.

You know your body better than anyone else, so be kind to it and you will learn to understand what it is truly asking. Be patient with yourself and this journey of change.

You are becoming the person you are, the person who has so much life to live for and to give to the world. You are taking steps towards freedom. It is tough, but the more you do, the closer you will be to freedom. Freedom from thinking about food all the time. Freedom from fear. Freedom from what other people might expect you to be.

Recovery is not linear, nor a perfect journey. Recovery is a battle, and you will become stronger and stronger along the way. Let other people in this battle, and it will become easier.

Remember the bigger picture. Remember why you want recovery and why you want to choose life over your eating disorder. Try to speak this motivation out loud now, as you approach the food.

Figure S1. The pictures of the virtual kitchen environment.

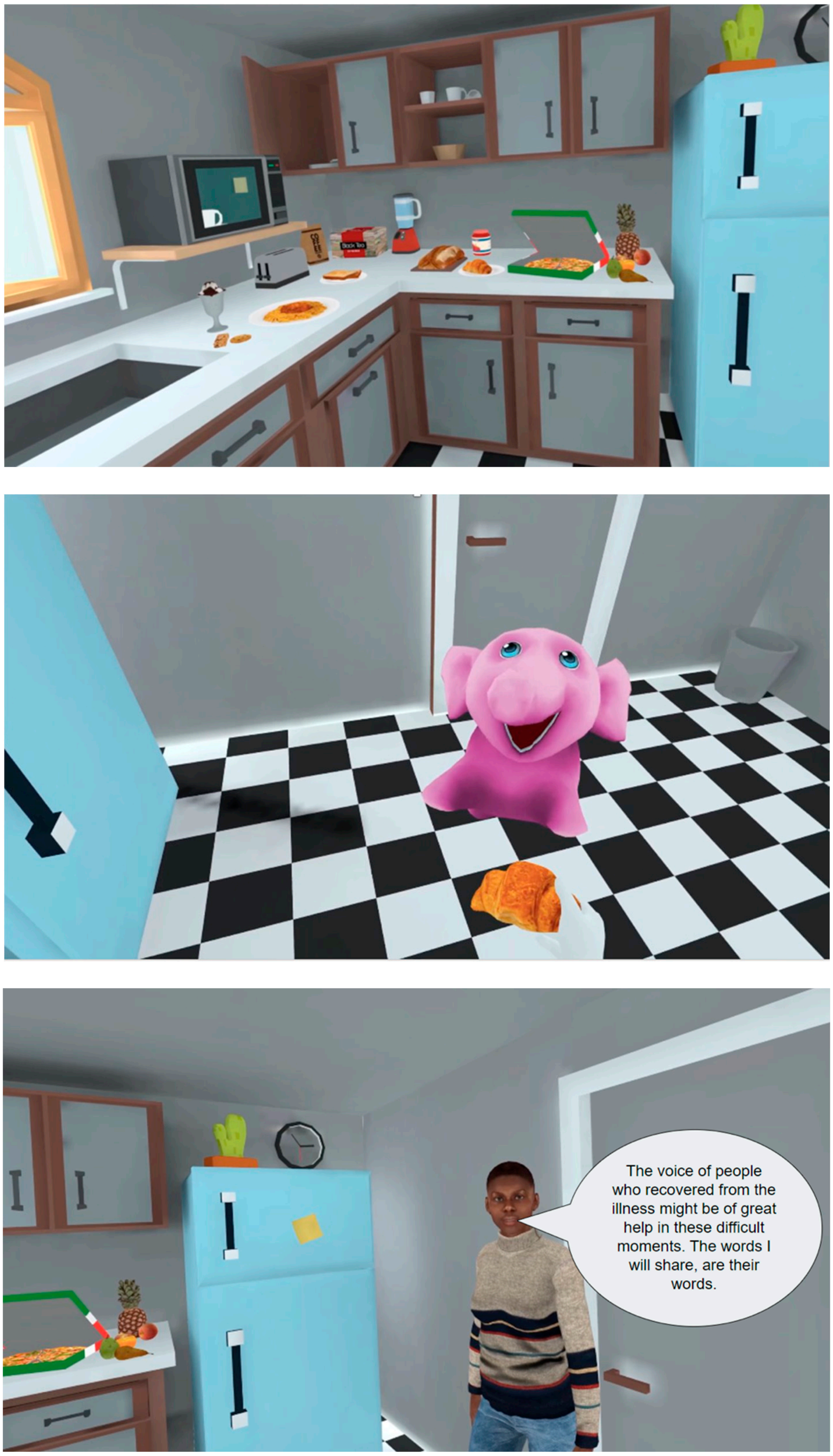

Figure S2. Selection of avatars available to participants

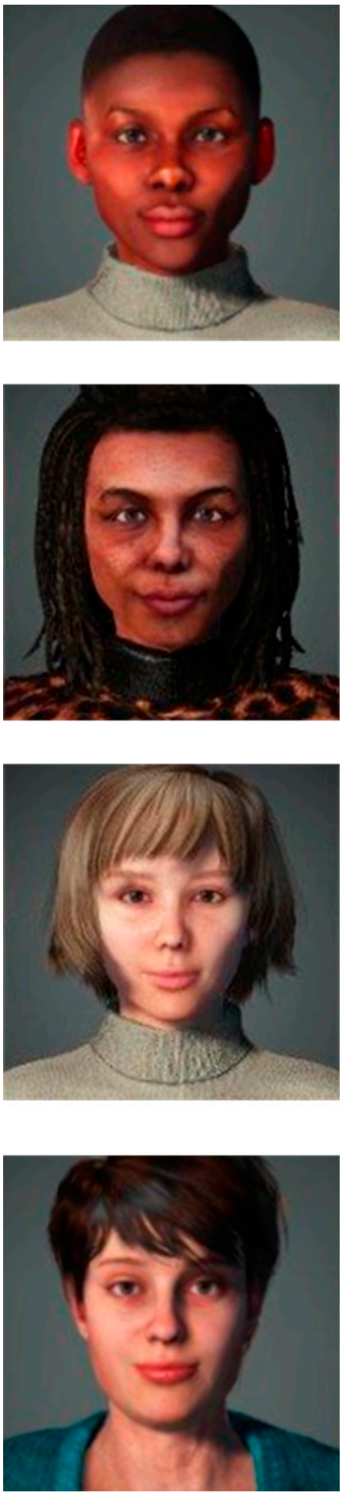

Figure S3. SPSS output for spearman rank-order correlations

(a) The variables of interest in the entire sample (n = 70)

Correlations

			FDS_Global	FDS_AnimalFlesh	FDS_PoorHygiene	FDS_HumanContamination	FDS_Mold	FDS_DecayingFruit	FDS_Fish	FDS_DecayingVegetables	FDS_LivingContaminants	PostExpD	EDEQ_Global	EDEQ_R	EDEQ_EAT	EDEQ_SHAPE	EDEQ_WEIGHT	BMI	VR_FoodGazes	VR_FoodTouches
Spearman's rho	FDS_Global	Correlation Coefficient	1.000	.498**	.544**	.600**	.785**	.703**	.815**	.714**	.577**	.157	.453**	.415**	.232	.526**	.433**	.145	.250*	-.218
		Sig. (2-tailed)	.	<.001	<.001	<.001	<.001	<.001	<.001	<.001	<.001	.204	<.001	<.001	.053	<.001	<.001	.232	.037	.070
		N	70	70	70	70	70	70	70	70	70	67	70	70	70	70	70	70	70	70
	FDS_AnimalFlesh	Correlation Coefficient	.498**	1.000	.287*	.266*	.166	-.010	.653**	.115	.234	.152	.279*	.262*	.196	.309**	.214	-.073	.001	-.085
		Sig. (2-tailed)	<.001	.	.016	.026	.170	.938	<.001	.345	.051	.219	.019	.029	.104	.009	.076	.546	.996	.486
		N	70	70	70	70	70	70	70	70	70	67	70	70	70	70	70	70	70	70
	FDS_PoorHygiene	Correlation Coefficient	.544**	.287*	1.000	.588**	.292*	.235	.308**	.352**	.224	.155	.270*	.340**	.064	.315**	.251*	-.029	.113	-.018
		Sig. (2-tailed)	<.001	.016	.	<.001	.014	.051	.009	.003	.062	.211	.024	.004	.596	.008	.036	.813	.351	.885
		N	70	70	70	70	70	70	70	70	70	67	70	70	70	70	70	70	70	70
	FDS_HumanContamination	Correlation Coefficient	.600**	.266*	.588**	1.000	.378**	.240*	.408**	.183	.339**	.115	.231	.271*	.078	.268*	.225	-.189	.119	-.063
		Sig. (2-tailed)	<.001	.026	<.001	.	.001	.045	<.001	.130	.004	.353	.055	.023	.522	.025	.061	.116	.327	.606
		N	70	70	70	70	70	70	70	70	70	67	70	70	70	70	70	70	70	70
	FDS_Mold	Correlation Coefficient	.785**	.166	.292*	.378**	1.000	.659**	.564**	.502**	.581**	.077	.422**	.397**	.196	.451**	.429**	.247*	.301*	-.250*
		Sig. (2-tailed)	<.001	.170	.014	.001	.	<.001	<.001	<.001	<.001	.535	<.001	<.001	.104	<.001	<.001	.039	.011	.037
		N	70	70	70	70	70	70	70	70	70	67	70	70	70	70	70	70	70	70
	FDS_DecayingFruit	Correlation Coefficient	.703**	-.010	.235	.240*	.659**	1.000	.442**	.704**	.289*	-.006	.238*	.103	.116	.296*	.290*	.265*	.363**	-.119
		Sig. (2-tailed)	<.001	.938	.051	.045	<.001	.	<.001	<.001	.015	.962	.047	.395	.337	.013	.015	.027	.002	.325
		N	70	70	70	70	70	70	70	70	70	67	70	70	70	70	70	70	70	70
	FDS_Fish	Correlation Coefficient	.815**	.653**	.308**	.408**	.564**	.442**	1.000	.488**	.380**	.216	.390**	.363**	.226	.441**	.362**	.080	.042	-.273*
		Sig. (2-tailed)	<.001	<.001	.009	<.001	<.001	<.001	.	<.001	.001	.080	<.001	.002	.060	<.001	.002	.508	.729	.022
		N	70	70	70	70	70	70	70	70	70	67	70	70	70	70	70	70	70	70
	FDS_DecayingVegetables	Correlation Coefficient	.714**	.115	.352**	.183	.502**	.704**	.488**	1.000	.345**	.052	.309**	.269*	.139	.413**	.320**	.219	.262*	-.128
		Sig. (2-tailed)	<.001	.345	.003	.130	<.001	<.001	<.001	.	.003	.675	.009	.024	.250	<.001	.007	.069	.028	.292
		N	70	70	70	70	70	70	70	70	70	67	70	70	70	70	70	70	70	70
	FDS_LivingContaminants	Correlation Coefficient	.577**	.234	.224	.339**	.581**	.289*	.380**	.345**	1.000	.031	.146	.207	.020	.218	.121	.215	.209	-.101
		Sig. (2-tailed)	<.001	.051	.062	.004	<.001	.015	.001	.003	.	.806	.228	.086	.869	.070	.317	.073	.083	.406
		N	70	70	70	70	70	70	70	70	70	67	70	70	70	70	70	70	70	70
	PostExpD	Correlation Coefficient	.157	.152	.155	.115	.077	-.006	.216	.052	.031	1.000	.655**	.623**	.577**	.603**	.529**	-.043	-.127	-.160
		Sig. (2-tailed)	.204	.219	.211	.353	.535	.962	.080	.675	.806	.	<.001	<.001	<.001	<.001	<.001	.732	.306	.196
		N	67	67	67	67	67	67	67	67	67	67	67	67	67	67	67	67	67	67
	EDEQ_Global	Correlation Coefficient	.453**	.279*	.270*	.231	.422**	.238*	.390**	.309**	.146	.655**	1.000	.843**	.854**	.905**	.910**	-.004	-.160	-.354**
		Sig. (2-tailed)	<.001	.019	.024	.055	<.001	.047	<.001	.009	.228	<.001	.	<.001	<.001	<.001	<.001	.977	.186	.003
		N	70	70	70	70	70	70	70	70	70	67	70	70	70	70	70	70	70	70
	EDEQ_R	Correlation Coefficient	.415**	.262*	.340**	.271*	.397**	.103	.363**	.269*	.207	.623**	.843**	1.000	.619**	.720**	.690**	-.134	-.224	-.278*
		Sig. (2-tailed)	<.001	.029	.004	.023	<.001	.395	.002	.024	.086	<.001	<.001	.	<.001	<.001	<.001	.268	.062	.020
		N	70	70	70	70	70	70	70	70	70	67	70	70	70	70	70	70	70	70
	EDEQ_EAT	Correlation Coefficient	.232	.196	.064	.078	.196	.116	.226	.139	.020	.577**	.854**	.619**	1.000	.654**	.680**	-.029	-.298*	-.281*
		Sig. (2-tailed)	.053	.104	.596	.522	.104	.337	.060	.250	.869	<.001	<.001	<.001	.	<.001	<.001	.809	.012	.019
		N	70	70	70	70	70	70	70	70	70	67	70	70	70	70	70	70	70	70
	EDEQ_SHAPE	Correlation Coefficient	.526**	.309**	.315**	.268*	.451**	.296*	.441**	.413**	.218	.603**	.905**	.720**	.654**	1.000	.883**	.091	-.015	-.310**
		Sig. (2-tailed)	<.001	.009	.008	.025	<.001	.013	<.001	<.001	.070	<.001	<.001	<.001	<.001	.	<.001	.454	.899	.009
		N	70	70	70	70	70	70	70	70	70	67	70	70	70	70	70	70	70	70
	EDEQ_WEIGHT	Correlation Coefficient	.433**	.214	.251*	.225	.429**	.290*	.362**	.320**	.121	.529**	.910**	.690**	.680**	.883**	1.000	.057	-.020	-.335**
		Sig. (2-tailed)	<.001	.076	.036	.061	<.001	.015	.002	.007	.317	<.001	<.001	<.001	<.001	<.001	.	.639	.871	.005
		N	70	70	70	70	70	70	70	70	70	67	70	70	70	70	70	70	70	70
	BMI	Correlation Coefficient	.145	-.073	-.029	-.189	.247*	.265*	.080	.219	.215	-.043	-.004	-.134	-.029	.091	.057	1.000	.268*	-.070
		Sig. (2-tailed)	.232	.546	.813	.116	.039	.027	.508	.069	.073	.732	.977	.268	.809	.454	.639	.	.025	.564
		N	70	70	70	70	70	70	70	70	70	67	70	70	70	70	70	70	70	70
	VR_FoodGazes	Correlation Coefficient	.250*	.001	.113	.119	.301*	.363**	.042	.262*	.209	-.127	-.160	-.224	-.298*	-.015	-.020	.268*	1.000	.333**
		Sig. (2-tailed)	.037	.996	.351	.327	.011	.002	.729	.028	.083	.306	.186	.062	.012	.899	.871	.025	.	.005
		N	70	70	70	70	70	70	70	70	70	67	70	70	70	70	70	70	70	70
	VR_FoodTouches	Correlation Coefficient	-.218	-.085	-.018	-.063	-.250*	-.119	-.273*	-.128	-.101	-.160	-.354**	-.278*	-.281*	-.310**	-.335**	-.070	.333**	1.000
		Sig. (2-tailed)	.070	.486	.885	.606	.037	.325	.022	.292	.406	.196	.003	.020	.019	.009	.005	.564	.005	.
		N	70	70	70	70	70	70	70	70	70	67	70	70	70	70	70	70	70	70

**. Correlation is significant at the 0.01 level (2-tailed).

*. Correlation is significant at the 0.05 level (2-tailed).

(b) The variables of interest in the only virtual kitchen scenario (n = 24)

Correlations

			FDS_Global	FDS_AnimalFlesh	FDS_PoorHygiene	FDS_HumanContamination	FDS_Mold	FDS_DecayingFruit	FDS_Fish	FDS_DecayingVegetables	FDS_LivingContaminants	PostExpD	EDEQ_Global	EDEQ_R	EDEQ_EAT	EDEQ_SHAPE	EDEQ_WEIGHT	BMI	VR_FoodGazes	VR_FoodTouches
Kitchen	FDS_Global	Correlation Coefficient	1.000	.623**	.488*	.488*	.760**	.609**	.866**	.544**	.361	.139	.533**	.428*	.321	.542**	.590**	.047	.110	-.357
		Sig. (2-tailed)	.	.001	.016	.016	<.001	.002	<.001	.006	.083	.517	.007	.037	.126	.006	.002	.828	.609	.087
		N	24	24	24	24	24	24	24	24	24	24	24	24	24	24	24	24	24	24
	FDS_AnimalFlesh	Correlation Coefficient	.623**	1.000	.364	.365	.344	-.089	.606**	.103	.364	.137	.289	.301	.102	.309	.265	-.179	.246	-.069
		Sig. (2-tailed)	.001	.	.081	.080	.100	.681	.002	.631	.080	.524	.170	.154	.635	.141	.211	.402	.247	.750
		N	24	24	24	24	24	24	24	24	24	24	24	24	24	24	24	24	24	24
	FDS_PoorHygiene	Correlation Coefficient	.488*	.364	1.000	.550**	.235	.067	.195	.208	.149	-.034	.260	.372	.097	.181	.186	-.204	.010	-.017
		Sig. (2-tailed)	.016	.081	.	.005	.269	.756	.362	.329	.488	.875	.219	.074	.651	.398	.383	.339	.965	.936
		N	24	24	24	24	24	24	24	24	24	24	24	24	24	24	24	24	24	24
	FDS_HumanContamination	Correlation Coefficient	.488*	.365	.550**	1.000	.272	.085	.371	-.154	.334	-.037	.094	.101	-.024	-.001	.171	-.266	-.054	-.015
		Sig. (2-tailed)	.016	.080	.005	.	.198	.693	.074	.472	.111	.862	.662	.638	.911	.997	.424	.210	.801	.945
		N	24	24	24	24	24	24	24	24	24	24	24	24	24	24	24	24	24	24
	FDS_Mold	Correlation Coefficient	.760**	.344	.235	.272	1.000	.486*	.741**	.247	.451*	.159	.633**	.569**	.320	.584**	.668**	.246	.038	-.552**
		Sig. (2-tailed)	<.001	.100	.269	.198	.	.016	<.001	.245	.027	.457	<.001	.004	.127	.003	<.001	.246	.859	.005
		N	24	24	24	24	24	24	24	24	24	24	24	24	24	24	24	24	24	24
	FDS_DecayingFruit	Correlation Coefficient	.609**	-.089	.067	.085	.486*	1.000	.499*	.626**	-.145	-.086	.229	-.021	.262	.258	.362	.302	.048	-.382
		Sig. (2-tailed)	.002	.681	.756	.693	.016	.	.013	.001	.500	.691	.281	.922	.216	.223	.082	.151	.822	.066
		N	24	24	24	24	24	24	24	24	24	24	24	24	24	24	24	24	24	24
	FDS_Fish	Correlation Coefficient	.866**	.606**	.195	.371	.741**	.499*	1.000	.300	.344	.237	.421*	.361	.210	.423*	.483*	.010	.007	-.489*
		Sig. (2-tailed)	<.001	.002	.362	.074	<.001	.013	.	.154	.100	.266	.040	.083	.325	.040	.017	.964	.976	.015
		N	24	24	24	24	24	24	24	24	24	24	24	24	24	24	24	24	24	24
	FDS_DecayingVegetables	Correlation Coefficient	.544**	.103	.208	-.154	.247	.626**	.300	1.000	-.101	.005	.364	.167	.310	.522**	.429*	.216	.114	-.056
		Sig. (2-tailed)	.006	.631	.329	.472	.245	.001	.154	.	.638	.980	.080	.434	.140	.009	.036	.311	.595	.796
		N	24	24	24	24	24	24	24	24	24	24	24	24	24	24	24	24	24	24
	FDS_LivingContaminants	Correlation Coefficient	.361	.364	.149	.334	.451*	-.145	.344	-.101	1.000	.086	.084	.229	-.128	.081	.104	.017	.140	-.244
		Sig. (2-tailed)	.083	.080	.488	.111	.027	.500	.100	.638	.	.689	.697	.283	.552	.705	.629	.937	.515	.251
		N	24	24	24	24	24	24	24	24	24	24	24	24	24	24	24	24	24	24
	PostExpD	Correlation Coefficient	.139	.137	-.034	-.037	.159	-.086	.237	.005	.086	1.000	.399	.467*	.382	.358	.165	-.038	-.269	-.140
		Sig. (2-tailed)	.517	.524	.875	.862	.457	.691	.266	.980	.689	.	.053	.022	.065	.086	.442	.859	.203	.513
		N	24	24	24	24	24	24	24	24	24	24	24	24	24	24	24	24	24	24
	EDEQ_Global	Correlation Coefficient	.533**	.289	.260	.094	.633**	.229	.421*	.364	.084	.399	1.000	.877**	.797**	.858**	.851**	.019	-.333	-.309
		Sig. (2-tailed)	.007	.170	.219	.662	<.001	.281	.040	.080	.697	.053	.	<.001	<.001	<.001	<.001	.929	.112	.141
		N	24	24	24	24	24	24	24	24	24	24	24	24	24	24	24	24	24	24
	EDEQ_R	Correlation Coefficient	.428*	.301	.372	.101	.569**	-.021	.361	.167	.229	.467*	.877**	1.000	.598**	.735**	.599**	-.125	-.377	-.269
		Sig. (2-tailed)	.037	.154	.074	.638	.004	.922	.083	.434	.283	.022	<.001	.	.002	<.001	.002	.559	.070	.203
		N	24	24	24	24	24	24	24	24	24	24	24	24	24	24	24	24	24	24
	EDEQ_EAT	Correlation Coefficient	.321	.102	.097	-.024	.320	.262	.210	.310	-.128	.382	.797**	.598**	1.000	.488*	.558**	-.079	-.422*	-.130
		Sig. (2-tailed)	.126	.635	.651	.911	.127	.216	.325	.140	.552	.065	<.001	.002	.	.016	.005	.714	.040	.545
		N	24	24	24	24	24	24	24	24	24	24	24	24	24	24	24	24	24	24
	EDEQ_SHAPE	Correlation Coefficient	.542**	.309	.181	-.001	.584**	.258	.423*	.522**	.081	.358	.858**	.735**	.488*	1.000	.864**	.208	-.140	-.276
		Sig. (2-tailed)	.006	.141	.398	.997	.003	.223	.040	.009	.705	.086	<.001	<.001	.016	.	<.001	.330	.514	.192
		N	24	24	24	24	24	24	24	24	24	24	24	24	24	24	24	24	24	24
	EDEQ_WEIGHT	Correlation Coefficient	.590**	.265	.186	.171	.668**	.362	.483*	.429*	.104	.165	.851**	.599**	.558**	.864**	1.000	.147	-.102	-.294
		Sig. (2-tailed)	.002	.211	.383	.424	<.001	.082	.017	.036	.629	.442	<.001	.002	.005	<.001	.	.493	.635	.163
		N	24	24	24	24	24	24	24	24	24	24	24	24	24	24	24	24	24	24
	BMI	Correlation Coefficient	.047	-.179	-.204	-.266	.246	.302	.010	.216	.017	-.038	.019	-.125	-.079	.208	.147	1.000	.115	-.357
		Sig. (2-tailed)	.828	.402	.339	.210	.246	.151	.964	.311	.937	.859	.929	.559	.714	.330	.493	.	.592	.087
		N	24	24	24	24	24	24	24	24	24	24	24	24	24	24	24	24	24	24
	VR_FoodGazes	Correlation Coefficient	.110	.246	.010	-.054	.038	.048	.007	.114	.140	-.269	-.333	-.377	-.422*	-.140	-.102	.115	1.000	.331
		Sig. (2-tailed)	.609	.247	.965	.801	.859	.822	.976	.595	.515	.203	.112	.070	.040	.514	.635	.592	.	.114
		N	24	24	24	24	24	24	24	24	24	24	24	24	24	24	24	24	24	24
	VR_FoodTouches	Correlation Coefficient	-.357	-.069	-.017	-.015	-.552**	-.382	-.489*	-.056	-.244	-.140	-.309	-.269	-.130	-.276	-.294	-.357	.331	1.000
		Sig. (2-tailed)	.087	.750	.936	.945	.005	.066	.015	.796	.251	.513	.141	.203	.545	.192	.163	.087	.114	.
		N	24	24	24	24	24	24	24	24	24	24	24	24	24	24	24	24	24	24

**. Correlation is significant at the 0.01 level (2-tailed).

*. Correlation is significant at the 0.05 level (2-tailed).

(c) The variables of interest in the virtual kitchen + pet scenario (n = 24)

Correlations

			FDS_Global	FDS_AnimalFlesh	FDS_PoorHygiene	FDS_HumanContamination	FDS_Mold	FDS_DecayingFruit	FDS_Fish	FDS_DecayingVegetables	FDS_LivingContaminants	PostExpD	EDEQ_Global	EDEQ_R	EDEQ_EAT	EDEQ_SHAPE	EDEQ_WEIGHT	BMI	VR_FoodGazes	VR_FoodTouches
Kitchen + Pet	FDS_Global	Correlation Coefficient	1.000	.376	.577**	.718**	.875**	.851**	.837**	.870**	.682**	.158	.334	.420*	-.050	.566**	.337	.095	.659**	.148
		Sig. (2-tailed)	.	.070	.003	<.001	<.001	<.001	<.001	<.001	<.001	.494	.111	.041	.818	.004	.107	.659	<.001	.491
		N	24	24	24	24	24	24	24	24	24	21	24	24	24	24	24	24	24	24
	FDS_AnimalFlesh	Correlation Coefficient	.376	1.000	.076	.228	.151	.035	.624**	.277	.266	-.109	.215	.245	.118	.252	.171	.151	.007	-.162
		Sig. (2-tailed)	.070	.	.725	.285	.482	.869	.001	.189	.209	.637	.314	.249	.582	.236	.424	.481	.973	.451
		N	24	24	24	24	24	24	24	24	24	21	24	24	24	24	24	24	24	24
	FDS_PoorHygiene	Correlation Coefficient	.577**	.076	1.000	.722**	.525**	.534**	.331	.435*	.308	.313	.298	.408*	-.099	.413*	.354	.133	.344	.251
		Sig. (2-tailed)	.003	.725	.	<.001	.008	.007	.115	.033	.143	.167	.158	.048	.645	.045	.090	.536	.100	.237
		N	24	24	24	24	24	24	24	24	24	21	24	24	24	24	24	24	24	24
	FDS_HumanContamination	Correlation Coefficient	.718**	.228	.722**	1.000	.592**	.506*	.537**	.485*	.331	.315	.477*	.441*	.098	.681**	.371	-.112	.404*	.204
		Sig. (2-tailed)	<.001	.285	<.001	.	.002	.012	.007	.016	.114	.165	.018	.031	.649	<.001	.074	.604	.050	.340
		N	24	24	24	24	24	24	24	24	24	21	24	24	24	24	24	24	24	24
	FDS_Mold	Correlation Coefficient	.875**	.151	.525**	.592**	1.000	.855**	.621**	.768**	.761**	.127	.258	.361	-.080	.478*	.328	.289	.744**	.100
		Sig. (2-tailed)	<.001	.482	.008	.002	.	<.001	.001	<.001	<.001	.584	.224	.083	.711	.018	.118	.171	<.001	.641
		N	24	24	24	24	24	24	24	24	24	21	24	24	24	24	24	24	24	24
	FDS_DecayingFruit	Correlation Coefficient	.851**	.035	.534**	.506*	.855**	1.000	.594**	.778**	.630**	.133	.183	.248	-.073	.412*	.251	.103	.642**	.135
		Sig. (2-tailed)	<.001	.869	.007	.012	<.001	.	.002	<.001	<.001	.566	.391	.243	.734	.045	.237	.631	<.001	.530
		N	24	24	24	24	24	24	24	24	24	21	24	24	24	24	24	24	24	24
	FDS_Fish	Correlation Coefficient	.837**	.624**	.331	.537**	.621**	.594**	1.000	.788**	.492*	-.120	.101	.163	-.151	.345	.099	.153	.426*	.117
		Sig. (2-tailed)	<.001	.001	.115	.007	.001	.002	.	<.001	.014	.603	.638	.447	.481	.099	.645	.477	.038	.587
		N	24	24	24	24	24	24	24	24	24	21	24	24	24	24	24	24	24	24
	FDS_DecayingVegetables	Correlation Coefficient	.870**	.277	.435*	.485*	.768**	.778**	.788**	1.000	.614**	.169	.305	.399	-.022	.545**	.361	.108	.560**	.009
		Sig. (2-tailed)	<.001	.189	.033	.016	<.001	<.001	<.001	.	.001	.464	.148	.053	.918	.006	.083	.616	.004	.967
		N	24	24	24	24	24	24	24	24	24	21	24	24	24	24	24	24	24	24
	FDS_LivingContaminants	Correlation Coefficient	.682**	.266	.308	.331	.761**	.630**	.492*	.614**	1.000	-.082	.083	.149	-.113	.340	.115	.447*	.497*	.082
		Sig. (2-tailed)	<.001	.209	.143	.114	<.001	<.001	.014	.001	.	.722	.700	.487	.599	.104	.592	.029	.013	.702
		N	24	24	24	24	24	24	24	24	24	21	24	24	24	24	24	24	24	24
	PostExpD	Correlation Coefficient	.158	-.109	.313	.315	.127	.133	-.120	.169	-.082	1.000	.802**	.731**	.699**	.670**	.698**	-.363	.166	-.149
		Sig. (2-tailed)	.494	.637	.167	.165	.584	.566	.603	.464	.722	.	<.001	<.001	<.001	<.001	<.001	.106	.473	.519
		N	21	21	21	21	21	21	21	21	21	21	21	21	21	21	21	21	21	21
	EDEQ_Global	Correlation Coefficient	.334	.215	.298	.477*	.258	.183	.101	.305	.083	.802**	1.000	.863**	.812**	.877**	.933**	-.419*	.092	-.474*
		Sig. (2-tailed)	.111	.314	.158	.018	.224	.391	.638	.148	.700	<.001	.	<.001	<.001	<.001	<.001	.041	.668	.019
		N	24	24	24	24	24	24	24	24	24	21	24	24	24	24	24	24	24	24
	EDEQ_R	Correlation Coefficient	.420*	.245	.408*	.441*	.361	.248	.163	.399	.149	.731**	.863**	1.000	.533**	.714**	.819**	-.296	.230	-.353
		Sig. (2-tailed)	.041	.249	.048	.031	.083	.243	.447	.053	.487	<.001	<.001	.	.007	<.001	<.001	.160	.279	.091
		N	24	24	24	24	24	24	24	24	24	21	24	24	24	24	24	24	24	24
	EDEQ_EAT	Correlation Coefficient	-.050	.118	-.099	.098	-.080	-.073	-.151	-.022	-.113	.699**	.812**	.533**	1.000	.592**	.705**	-.419*	-.137	-.621**
		Sig. (2-tailed)	.818	.582	.645	.649	.711	.734	.481	.918	.599	<.001	<.001	.007	.	.002	<.001	.042	.525	.001
		N	24	24	24	24	24	24	24	24	24	21	24	24	24	24	24	24	24	24
	EDEQ_SHAPE	Correlation Coefficient	.566**	.252	.413*	.681**	.478*	.412*	.345	.545**	.340	.670**	.877**	.714**	.592**	1.000	.809**	-.240	.242	-.220
		Sig. (2-tailed)	.004	.236	.045	<.001	.018	.045	.099	.006	.104	<.001	<.001	<.001	.002	.	<.001	.258	.255	.302
		N	24	24	24	24	24	24	24	24	24	21	24	24	24	24	24	24	24	24
	EDEQ_WEIGHT	Correlation Coefficient	.337	.171	.354	.371	.328	.251	.099	.361	.115	.698**	.933**	.819**	.705**	.809**	1.000	-.318	.122	-.490*
		Sig. (2-tailed)	.107	.424	.090	.074	.118	.237	.645	.083	.592	<.001	<.001	<.001	<.001	<.001	.	.130	.571	.015
		N	24	24	24	24	24	24	24	24	24	21	24	24	24	24	24	24	24	24
	BMI	Correlation Coefficient	.095	.151	.133	-.112	.289	.103	.153	.108	.447*	-.363	-.419*	-.296	-.419*	-.240	-.318	1.000	.354	.335
		Sig. (2-tailed)	.659	.481	.536	.604	.171	.631	.477	.616	.029	.106	.041	.160	.042	.258	.130	.	.090	.110
		N	24	24	24	24	24	24	24	24	24	21	24	24	24	24	24	24	24	24
	VR_FoodGazes	Correlation Coefficient	.659**	.007	.344	.404*	.744**	.642**	.426*	.560**	.497*	.166	.092	.230	-.137	.242	.122	.354	1.000	.372
		Sig. (2-tailed)	<.001	.973	.100	.050	<.001	<.001	.038	.004	.013	.473	.668	.279	.525	.255	.571	.090	.	.073
		N	24	24	24	24	24	24	24	24	24	21	24	24	24	24	24	24	24	24
	VR_FoodTouches	Correlation Coefficient	.148	-.162	.251	.204	.100	.135	.117	.009	.082	-.149	-.474*	-.353	-.621**	-.220	-.490*	.335	.372	1.000
		Sig. (2-tailed)	.491	.451	.237	.340	.641	.530	.587	.967	.702	.519	.019	.091	.001	.302	.015	.110	.073	.
		N	24	24	24	24	24	24	24	24	24	21	24	24	24	24	24	24	24	24

**. Correlation is significant at the 0.01 level (2-tailed).
*. Correlation is significant at the 0.05 level (2-tailed).

(d) The variables of interest in the virtual kitchen + avatar scenario (n = 22)

Correlations

			FDS_Global	FDS_AnimalFlesh	FDS_PoorHygiene	FDS_HumanContamination	FDS_Mold	FDS_DecayingFruit	FDS_Fish	FDS_DecayingVegetables	FDS_LivingContaminants	PostExpD	EDEQ_Global	EDEQ_R	EDEQ_EAT	EDEQ_SHAPE	EDEQ_WEIGHT	BMI	VR_FoodGazes	VR_FoodTouches
Kitchen + Avatar	FDS_Global	Correlation Coefficient	1.000	.468*	.659**	.532*	.683**	.602**	.650**	.731**	.608**	.190	.451*	.398	.472*	.463*	.369	.287	.020	-.326
		Sig. (2-tailed)	.	.028	<.001	.011	<.001	.003	.001	<.001	.003	.397	.035	.067	.027	.030	.091	.195	.930	.139
		N	22	22	22	22	22	22	22	22	22	22	22	22	22	22	22	22	22	22
	FDS_AnimalFlesh	Correlation Coefficient	.468*	1.000	.474*	.188	.025	-.037	.665**	.027	-.026	.386	.266	.221	.376	.338	.177	-.084	-.224	-.014
		Sig. (2-tailed)	.028	.	.026	.402	.913	.872	<.001	.907	.910	.076	.231	.322	.085	.124	.431	.711	.317	.951
		N	22	22	22	22	22	22	22	22	22	22	22	22	22	22	22	22	22	22
	FDS_PoorHygiene	Correlation Coefficient	.659**	.474*	1.000	.597**	.177	.019	.534*	.575**	.257	.226	.250	.291	.209	.331	.212	-.091	-.065	-.397
		Sig. (2-tailed)	<.001	.026	.	.003	.431	.933	.010	.005	.248	.312	.262	.189	.351	.132	.343	.688	.774	.067
		N	22	22	22	22	22	22	22	22	22	22	22	22	22	22	22	22	22	22
	FDS_HumanContamination	Correlation Coefficient	.532*	.188	.597**	1.000	.373	.108	.300	.230	.375	-.060	.169	.347	.247	.150	.107	-.016	.021	-.442*
		Sig. (2-tailed)	.011	.402	.003	.	.087	.631	.174	.302	.086	.790	.452	.113	.267	.507	.635	.944	.926	.040
		N	22	22	22	22	22	22	22	22	22	22	22	22	22	22	22	22	22	22
	FDS_Mold	Correlation Coefficient	.683**	.025	.177	.373	1.000	.589**	.338	.477*	.595**	-.031	.334	.252	.345	.295	.240	.212	.086	-.325
		Sig. (2-tailed)	<.001	.913	.431	.087	.	.004	.123	.025	.003	.890	.129	.258	.115	.182	.283	.344	.704	.140
		N	22	22	22	22	22	22	22	22	22	22	22	22	22	22	22	22	22	22
	FDS_DecayingFruit	Correlation Coefficient	.602**	-.037	.019	.108	.589**	1.000	.165	.505*	.409	-.001	.188	.043	.116	.178	.220	.557**	.472*	-.110
		Sig. (2-tailed)	.003	.872	.933	.631	.004	.	.463	.017	.059	.995	.402	.848	.608	.429	.325	.007	.027	.627
		N	22	22	22	22	22	22	22	22	22	22	22	22	22	22	22	22	22	22
	FDS_Fish	Correlation Coefficient	.650**	.665**	.534*	.300	.338	.165	1.000	.456*	.206	.539**	.604**	.569**	.688**	.525*	.463*	.190	-.379	-.459*
		Sig. (2-tailed)	.001	<.001	.010	.174	.123	.463	.	.033	.358	.010	.003	.006	<.001	.012	.030	.397	.082	.032
		N	22	22	22	22	22	22	22	22	22	22	22	22	22	22	22	22	22	22
	FDS_DecayingVegetables	Correlation Coefficient	.731**	.027	.575**	.230	.477*	.505*	.456*	1.000	.566**	.146	.380	.324	.269	.345	.251	.248	.008	-.294
		Sig. (2-tailed)	<.001	.907	.005	.302	.025	.017	.033	.	.006	.517	.081	.142	.226	.116	.260	.266	.972	.184
		N	22	22	22	22	22	22	22	22	22	22	22	22	22	22	22	22	22	22
	FDS_LivingContaminants	Correlation Coefficient	.608**	-.026	.257	.375	.595**	.409	.206	.566**	1.000	.082	.245	.312	.282	.211	.161	.310	.035	-.190
		Sig. (2-tailed)	.003	.910	.248	.086	.003	.059	.358	.006	.	.716	.271	.158	.203	.346	.473	.161	.879	.398
		N	22	22	22	22	22	22	22	22	22	22	22	22	22	22	22	22	22	22
	PostExpD	Correlation Coefficient	.190	.386	.226	-.060	-.031	-.001	.539**	.146	.082	1.000	.776**	.725**	.716**	.740**	.733**	.238	-.260	-.195
		Sig. (2-tailed)	.397	.076	.312	.790	.890	.995	.010	.517	.716	.	<.001	<.001	<.001	<.001	<.001	.287	.243	.383
		N	22	22	22	22	22	22	22	22	22	22	22	22	22	22	22	22	22	22
	EDEQ_Global	Correlation Coefficient	.451*	.266	.250	.169	.334	.188	.604**	.380	.245	.776**	1.000	.790**	.934**	.925**	.908**	.280	-.229	-.401
		Sig. (2-tailed)	.035	.231	.262	.452	.129	.402	.003	.081	.271	<.001	.	<.001	<.001	<.001	<.001	.208	.306	.065
		N	22	22	22	22	22	22	22	22	22	22	22	22	22	22	22	22	22	22
	EDEQ_R	Correlation Coefficient	.398	.221	.291	.347	.252	.043	.569**	.324	.312	.725**	.790**	1.000	.757**	.649**	.654**	.029	-.432*	-.300
		Sig. (2-tailed)	.067	.322	.189	.113	.258	.848	.006	.142	.158	<.001	<.001	.	<.001	.001	<.001	.898	.044	.175
		N	22	22	22	22	22	22	22	22	22	22	22	22	22	22	22	22	22	22
	EDEQ_EAT	Correlation Coefficient	.472*	.376	.209	.247	.345	.116	.688**	.269	.282	.716**	.934**	.757**	1.000	.817**	.806**	.338	-.303	-.374
		Sig. (2-tailed)	.027	.085	.351	.267	.115	.608	<.001	.226	.203	<.001	<.001	<.001	.	<.001	<.001	.124	.171	.087
		N	22	22	22	22	22	22	22	22	22	22	22	22	22	22	22	22	22	22
	EDEQ_SHAPE	Correlation Coefficient	.463*	.338	.331	.150	.295	.178	.525*	.345	.211	.740**	.925**	.649**	.817**	1.000	.916**	.175	-.150	-.376
		Sig. (2-tailed)	.030	.124	.132	.507	.182	.429	.012	.116	.346	<.001	<.001	.001	<.001	.	<.001	.435	.505	.085
		N	22	22	22	22	22	22	22	22	22	22	22	22	22	22	22	22	22	22
	EDEQ_WEIGHT	Correlation Coefficient	.369	.177	.212	.107	.240	.220	.463*	.251	.161	.733**	.908**	.654**	.806**	.916**	1.000	.324	-.042	-.324
		Sig. (2-tailed)	.091	.431	.343	.635	.283	.325	.030	.260	.473	<.001	<.001	<.001	<.001	<.001	.	.141	.851	.141
		N	22	22	22	22	22	22	22	22	22	22	22	22	22	22	22	22	22	22
	BMI	Correlation Coefficient	.287	-.084	-.091	-.016	.212	.557**	.190	.248	.310	.238	.280	.029	.338	.175	.324	1.000	.249	-.213
		Sig. (2-tailed)	.195	.711	.688	.944	.344	.007	.397	.266	.161	.287	.208	.898	.124	.435	.141	.	.265	.342
		N	22	22	22	22	22	22	22	22	22	22	22	22	22	22	22	22	22	22
	VR_FoodGazes	Correlation Coefficient	.020	-.224	-.065	.021	.086	.472*	-.379	.008	.035	-.260	-.229	-.432*	-.303	-.150	-.042	.249	1.000	.278
		Sig. (2-tailed)	.930	.317	.774	.926	.704	.027	.082	.972	.879	.243	.306	.044	.171	.505	.851	.265	.	.210
		N	22	22	22	22	22	22	22	22	22	22	22	22	22	22	22	22	22	22
	VR_FoodTouches	Correlation Coefficient	-.326	-.014	-.397	-.442*	-.325	-.110	-.459*	-.294	-.190	-.195	-.401	-.300	-.374	-.376	-.324	-.213	.278	1.000
		Sig. (2-tailed)	.139	.951	.067	.040	.140	.627	.032	.184	.398	.383	.065	.175	.087	.085	.141	.342	.210	.
		N	22	22	22	22	22	22	22	22	22	22	22	22	22	22	22	22	22	22

*. Correlation is significant at the 0.05 level (2-tailed).
**. Correlation is significant at the 0.01 level (2-tailed).

Figure S4. The scatter plot figures.

(a) The scatter plots of food disgust sensitivity vs eating disorder severity variables (EDE-Q and BMI)

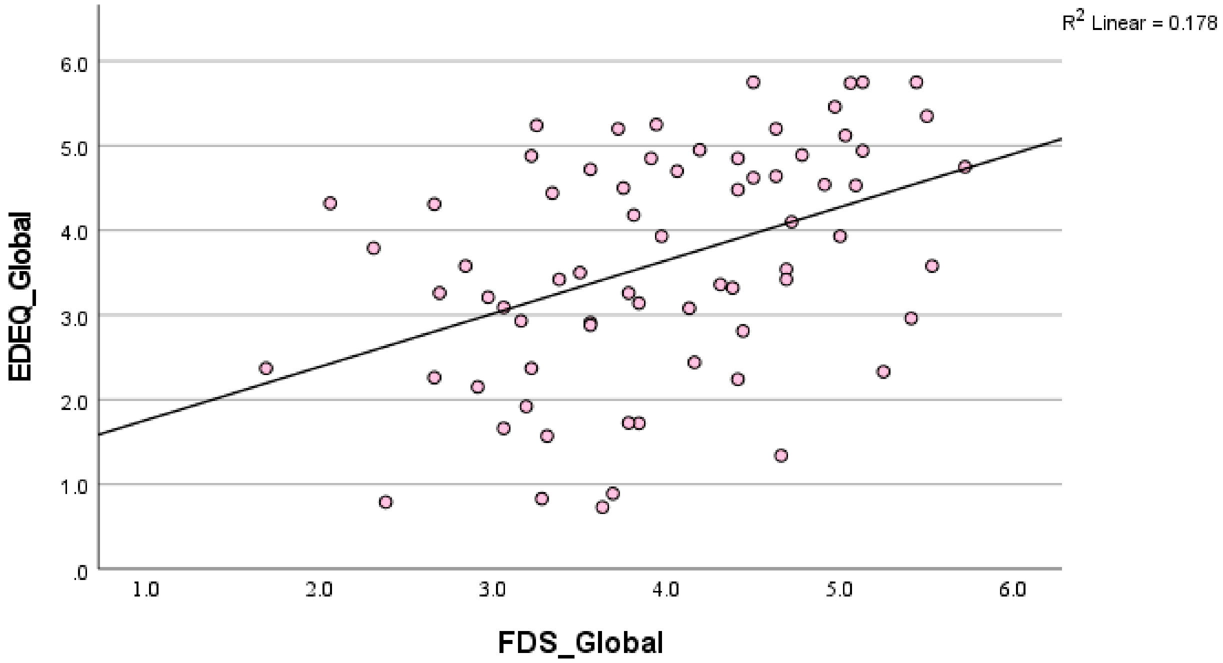

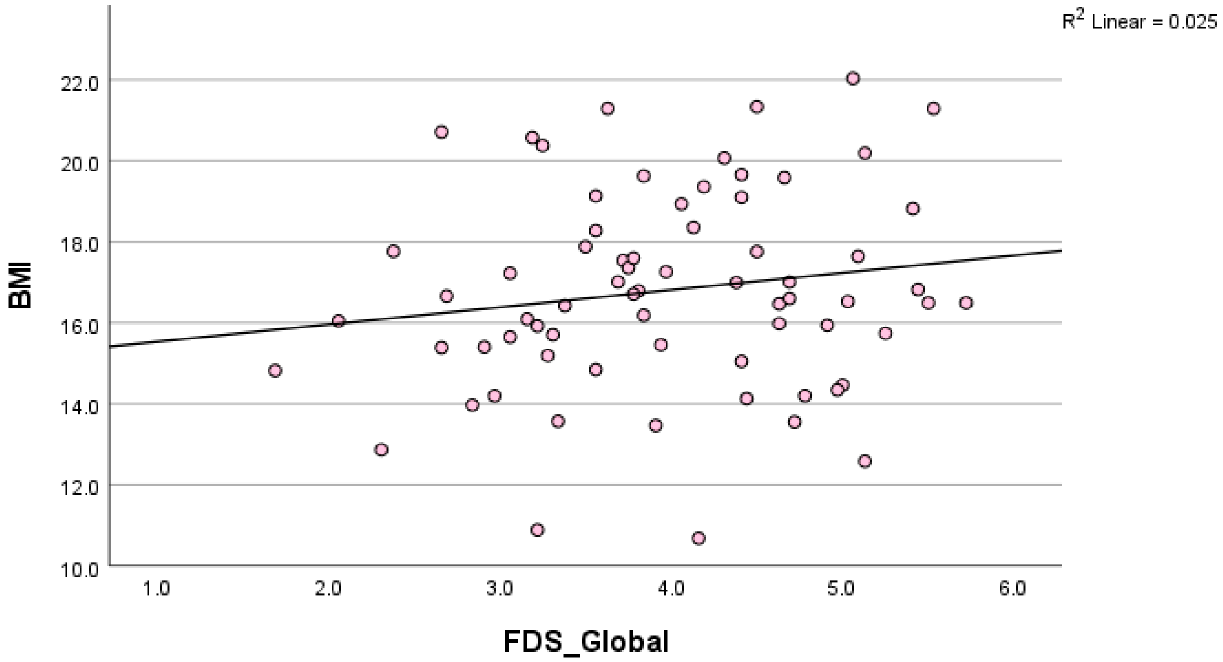

(b) The scatter plots of food disgust sensitivity vs food interaction measures in the VR (eye gazes and touches towards virtual foods)

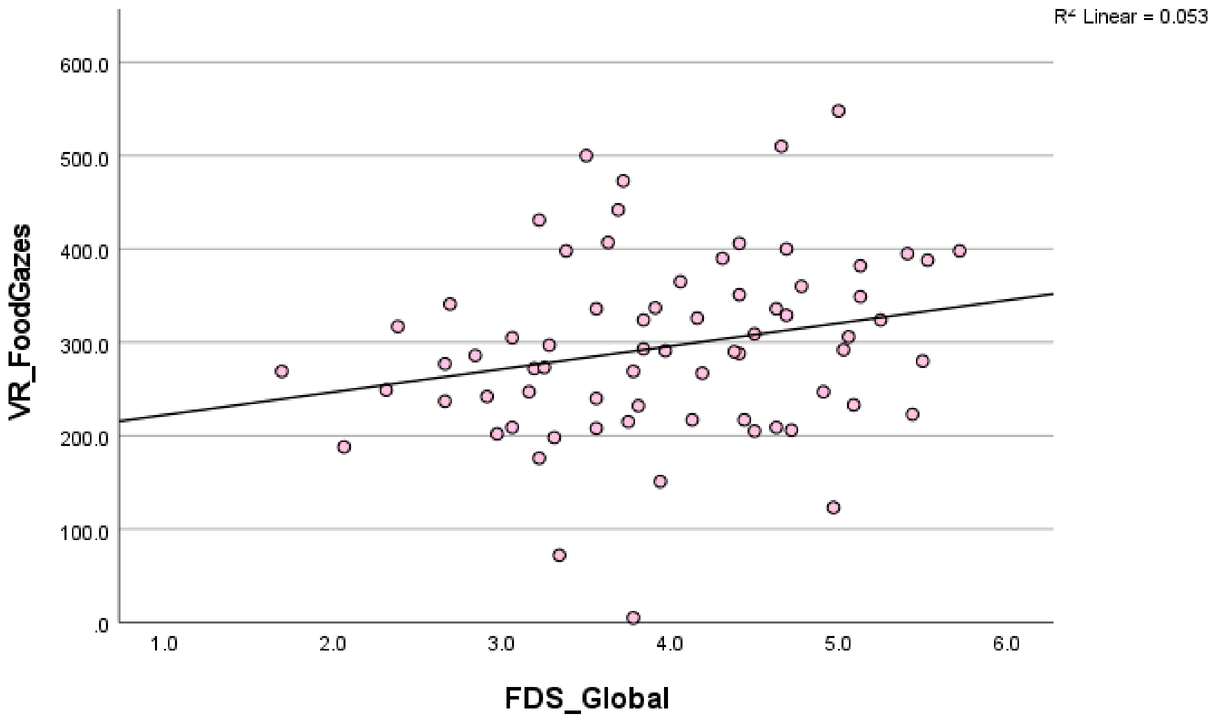

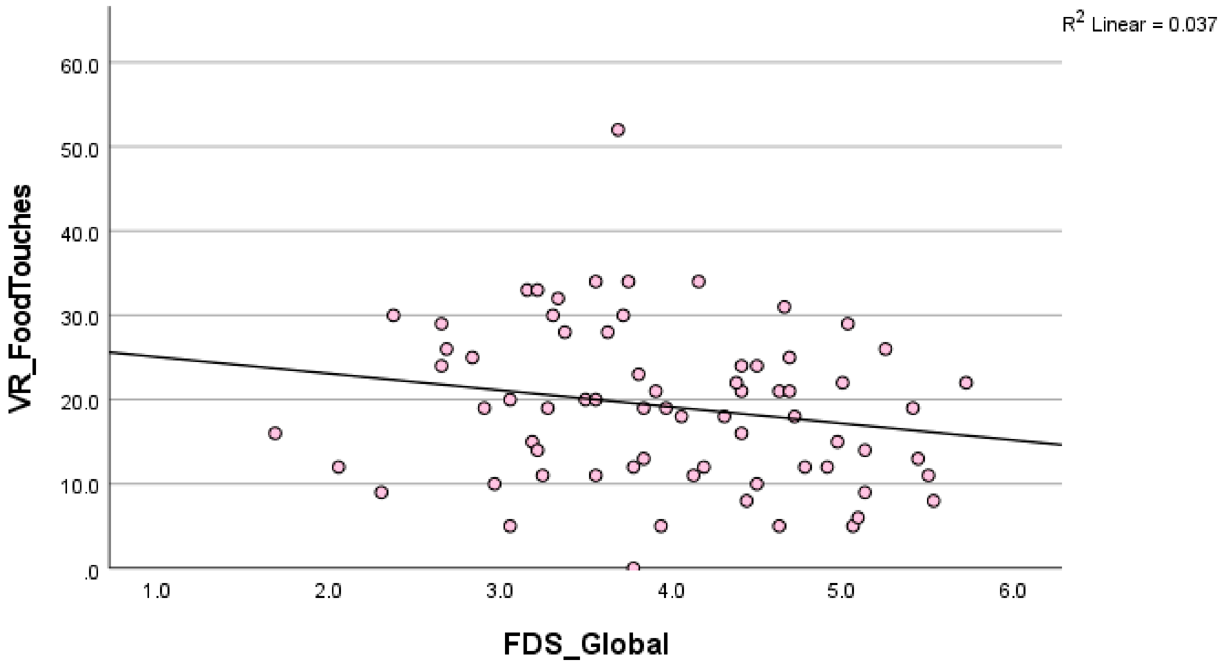

(c) The scatter plots of momentary disgust reactions towards virtual foods with high-calorie content vs eating disorder severity variables (EDE-Q and BMI)

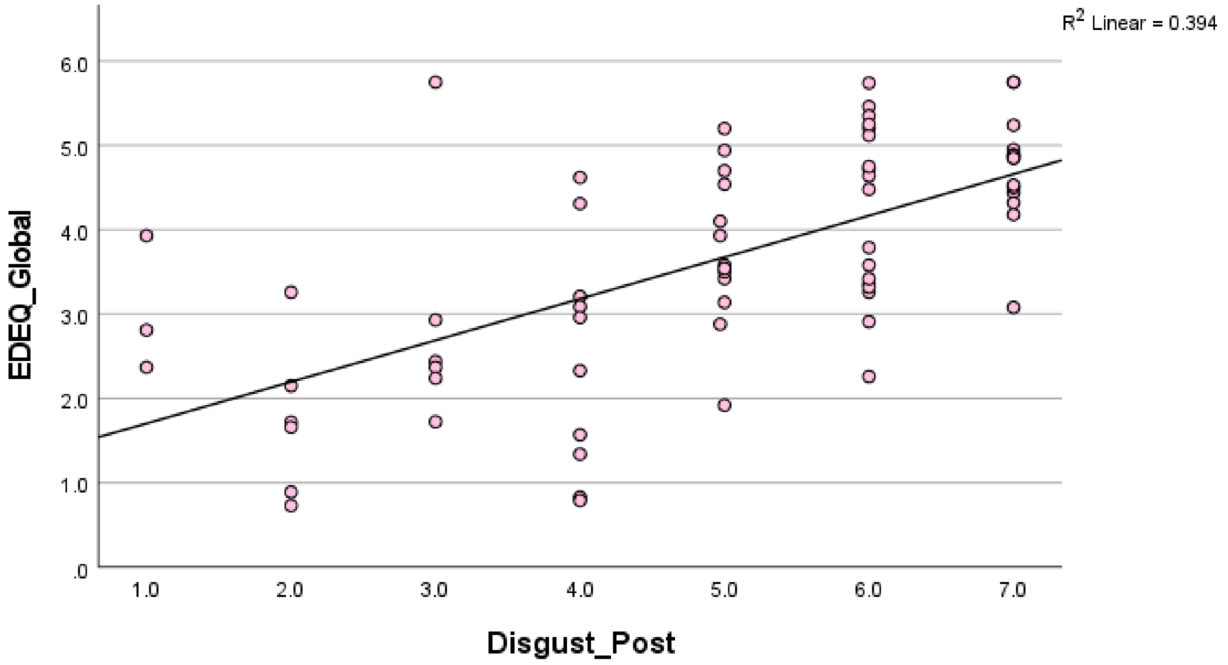

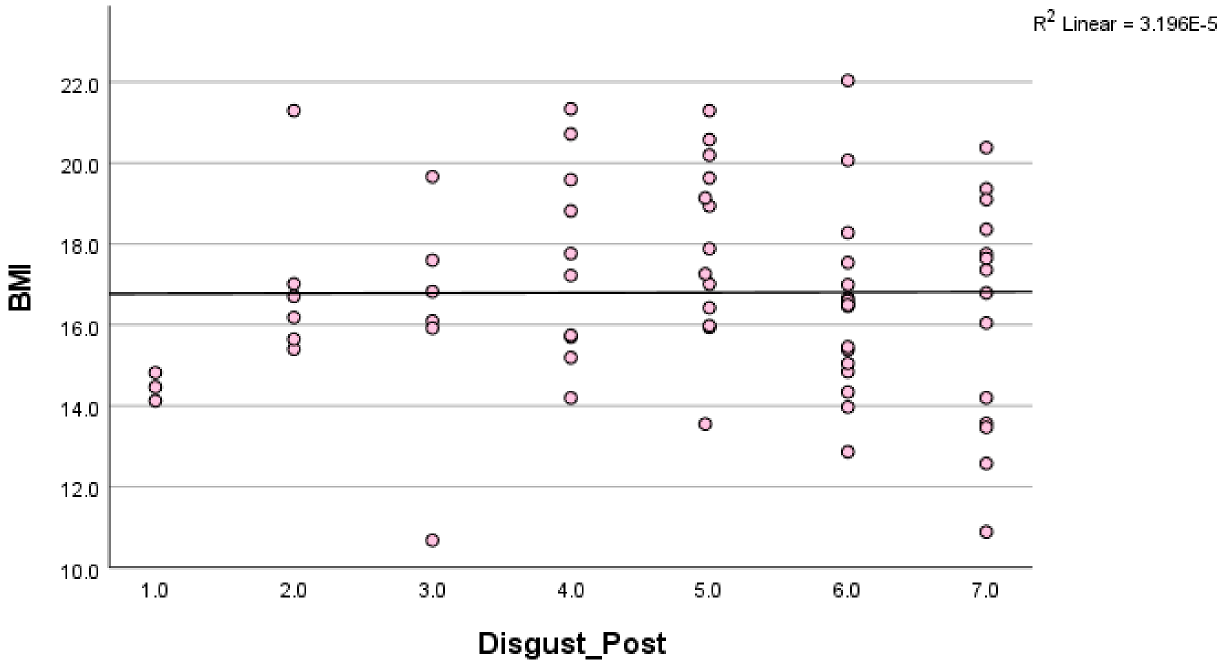

(d) The scatter plots of momentary disgust reactions towards virtual foods with high-calorie content vs food interaction measures in the VR (eye gazes and touches towards virtual foods)

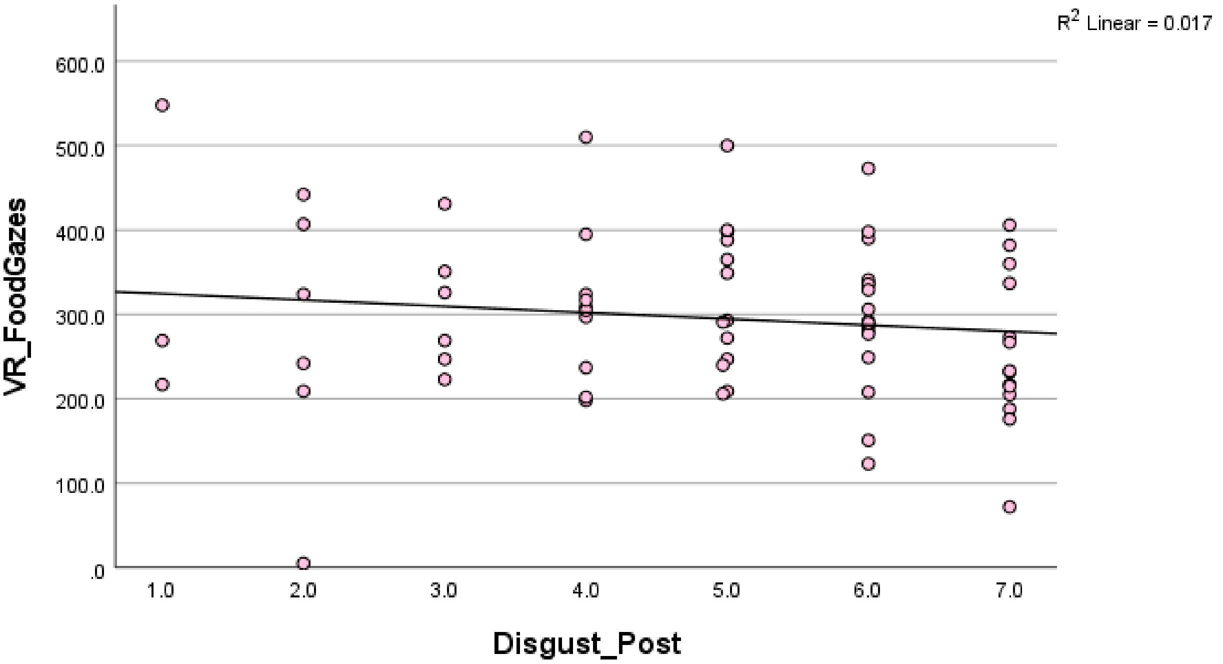

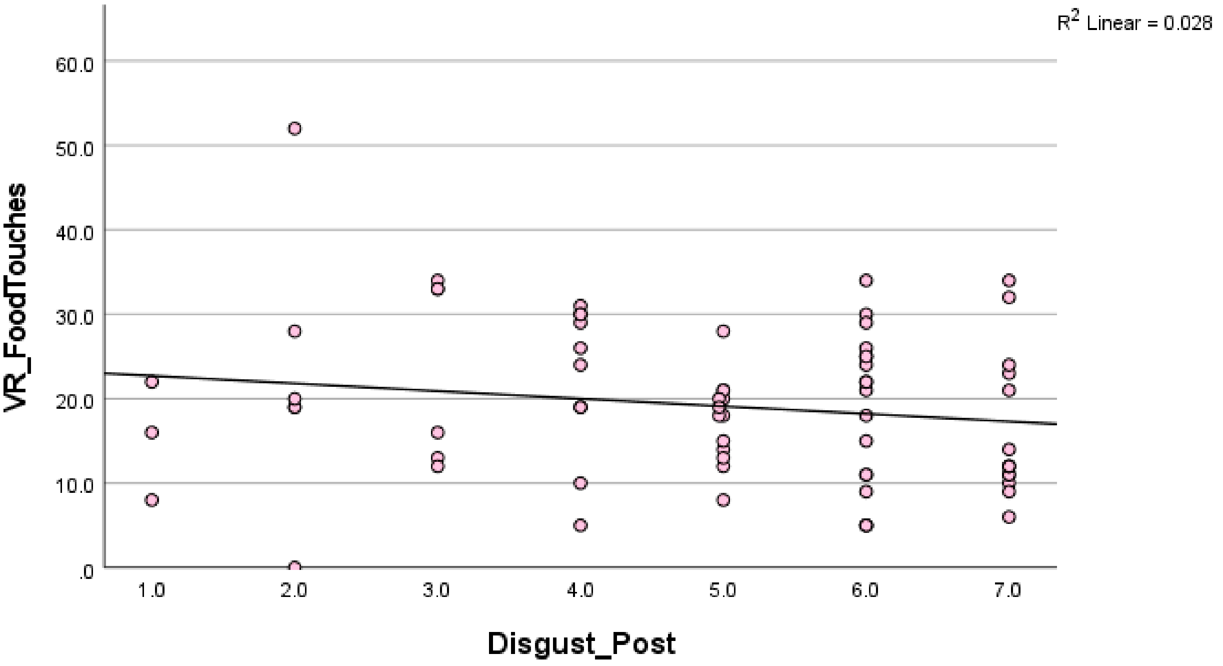

Supplement: Supplementary file 1 [file nutrients-15-04443-s001.zip › nutrients-2637300-supplementary.pdf]
